# A clinical and safety review of paracetamol and ibuprofen in children

**DOI:** 10.1007/s10787-016-0302-3

**Published:** 2017-01-06

**Authors:** Dipak J. Kanabar

**Affiliations:** Evelina Children’s Hospital, London, SE1 7EH UK

**Keywords:** Ibuprofen, Paracetamol, Acetaminophen, Safety, Children

## Abstract

The antipyretic analgesics, paracetamol, and non-steroidal anti-inflammatory agents NSAIDs are one of the most widely used classes of medications in children. The aim of this review is to determine if there are any clinically relevant differences in safety between ibuprofen and paracetamol that may recommend one agent over the other in the management of fever and discomfort in children older than 3 months of age.

## Methods: a review of the current literature

Pre-school children suffer frequent episodes of illness leading to more primary care consultations than any other age group (McCormick et al. 1991). The most common reasons are coughs, colds, earaches and fevers (Hay and Heron 2005), with fever as the primary presentation for these illnesses.

The underlying cause of childhood fever is generally benign, and fever has a beneficial effect in terms of fighting infection (Sullivan and Farrar 2011). However, fever can cause distress and discomfort in children, leading to a high degree of parental concern. For febrile children without any indication of a serious underlying condition (‘low-risk’ fever), and key among the updated recommendations, is the need to treat symptoms of the fever with a focus on comforting the child, rather than on achieving normothermia.

National guidelines recommend home management (NICE 2013; Sullivan and Farrar 2011; Chiappini et al. 2012; Oteman et al. 2008) and childhood fever associated with discomfort or pain can be easily treated with over-the-counter (OTC) antipyretic (and analgesic) agents such as paracetamol (acetaminophen) and ibuprofen. Both agents are generally well tolerated and given equal status in both national and international guidelines. Current advice in the UK also states that paracetamol should be used to treat post-immunization fever in babies after their meningococcal B injections at age 2 and 4 months; in this instance, ibuprofen is not currently recommended.

In light of some of the above recommendations, parents of young children and health-care professionals may perceive paracetamol as being safer than ibuprofen. For pharmacists, who are likely to be safety driven, this perception may be fuelled by a lack of differentiation between the gastrointestinal (GI) safety profile of OTC and prescription (Rx) doses of NSAIDs. Most pharmacists also consider NSAIDs as a class, rather than isolating individual analgesics and assessing their GI safety profiles. Combined, this behaviour encourages the perception of ibuprofen having a poorer GI safety profile than paracetamol rather than being on a sliding scale of risk.

For paediatrics, first-line treatment for mild-to-moderate pain is either ibuprofen or paracetamol. Aspirin should not be given to children under 16 years unless on the advice of a doctor, because there is a very small risk that children can develop a condition called Reye’s syndrome if they are given aspirin when they have a viral illness. If pain relief is inadequate, second-line treatment is switching from one agent to the other, and third-line is treatment is to alternate between the two. Preliminary results of ongoing research into the prescribing habits of doctors and recommendations made by pharmacists have identified the following trends:

- Ibuprofen and paracetamol are the commonly prescribed analgesics.
- For fever, paracetamol is the most commonly prescribed for both paediatric and adult patients.
- For pain, ibuprofen is the most commonly prescribed for paediatrics (followed by paracetamol); however they share the top spot for adult patients.
- For inflammation, ibuprofen is the most commonly prescribed for both paediatric and adult patients.

When ibuprofen is administered at therapeutic doses in children of up to 10 mg/kg body weight every 6–8 h the possible adverse events are, as for other NSAIDs related to inhibition of cyclo-oxygenase (COX-1 and COX-2) and prostaglandin (PG) pathways, gastrointestinal bleeding, renal impairment, asthma and hepatic toxicity. Rainsford et al. (1997) have reviewed the safety of paracetamol and ibuprofen administered in adults at therapeutic dosages. The authors concluded that both agents were safe as used in clinical trials, and that there are no statistically significant differences between paracetamol and ibuprofen in reports of adverse events in any organ system, irrespective of the type or frequency of event. Across a range of clinical studies in which either ibuprofen or paracetamol were treatments of primary interest, the overall percentage of patients having a minor adverse event was about 10% with paracetamol compared with 8% with ibuprofen, for drug exposure up to 30 days, which is not unexpected for events that are monitored prospectively. However, with such ubiquitous usage of both agents, the increased reporting of rare or idiosyncratic side effects and consequences of unintentional (or intentional) overdosing is a likely occurrence.

## Safety

**Fig. 1:**
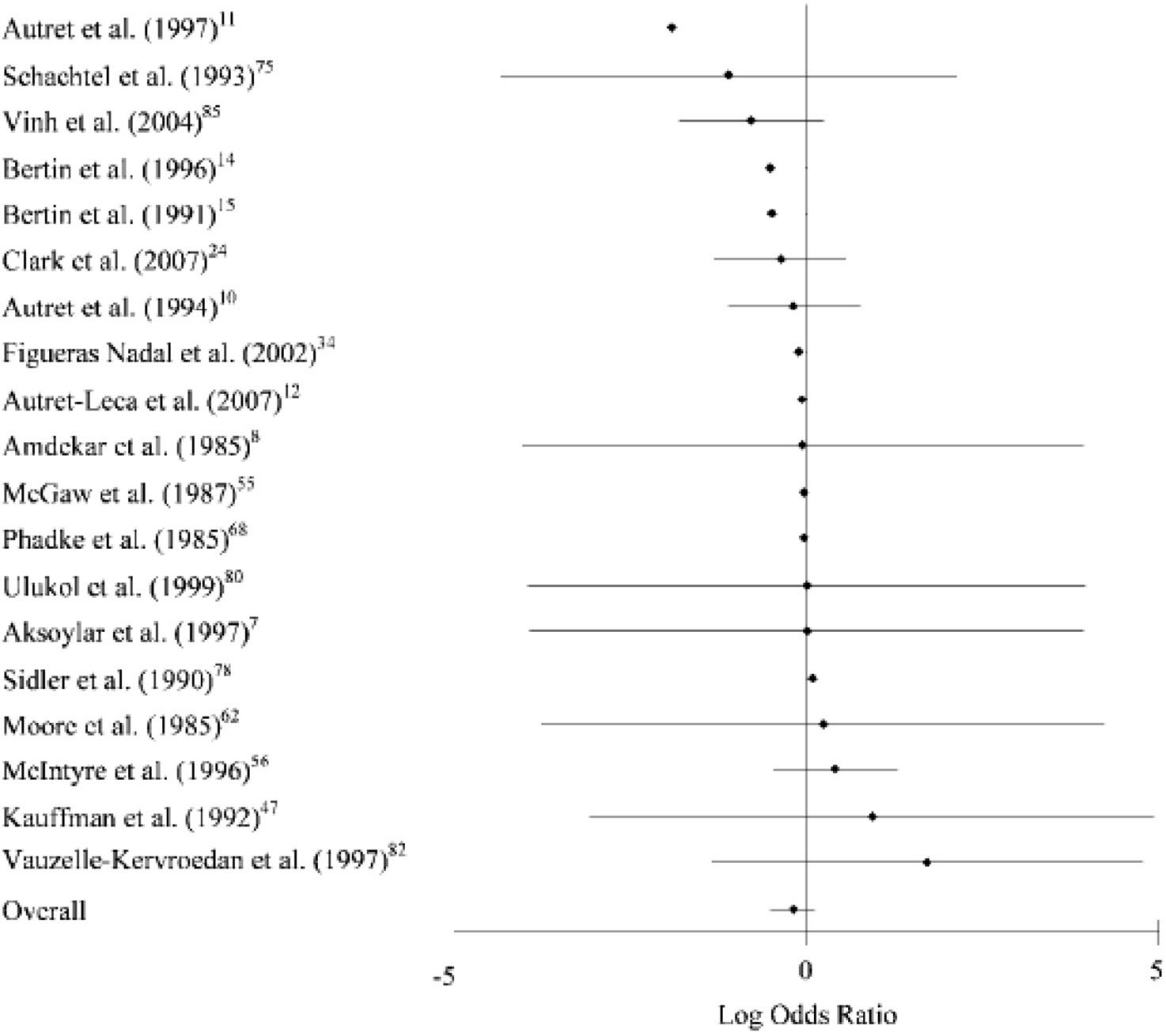
Forest plot of amended log odds ratios comparing proportion of children experiencing at least one adverse event for paracetamol versus ibuprofen. Negative numbers denote that the odds rations in the paracetamol treatment group are lower than the ibuprofen groups (reproduced with kind permission from Pierce and Voss 2010)

Safety is clearly a primary consideration in the choice of antipyretic, and both ibuprofen and paracetamol have been associated with safety issues, not all of which appear to be evidence based. Overall, ibuprofen and paracetamol are considered to have similar safety and tolerability profiles in paediatric fever, and this has been confirmed in meta-analyses (Southey et al. 2009; Pierce and Voss 2010). For example, a recent meta-analysis including 19 evaluable studies found no significant difference between the two agents in the incidence of adverse events in paediatric patients (0.82; 95% CI 0.60–1.12) (Pierce and Voss 2010) (Fig. 1).

However, a number of specific safety issues are often raised for both agents, which may impact on recommendations and prescribing practice. The question arises as to whether these concerns are evidence based, or have arisen due to medical ‘myths’ or ‘dogma.’

## Specific safety issues

### Gastrointestinal effects

The mechanism of acute NSAID-induced upper gastrointestinal complications (UGIC’s) is likely due to the combined result of topical effects and inhibition of both COX-1 and COX-2. Inhibition of COX-1 reduces microvascular blood flow; topical effects are due to lipid solubility and low p*K*a which renders NSAIDs a detergent and an uncoupler of mitochondrial oxidative phosphorylation: all these effects are dose dependent. The local effects of COX-2 inhibition are uncertain.

Dose-dependent gastrointestinal (GI) toxicity (e.g. bleeding) in association with NSAID treatment in adults is well documented in ‘at-risk’ patients (Bjarnason 2013). In adults, the rate of gastrointestinal symptoms associated with low-dose ibuprofen (1200 mg/day) is similar to that reported with paracetamol and placebo, but less than with aspirin (Moore et al. 1999). One study in which approximately 2000 adult patients taking NSAIDs were compared with 11,500 controls in a nested case–control analysis showed that the average relative risk of significant bleeding with NSAID is 3.0. There is, however, a hierarchy of risk among NSAIDs, with ibuprofen ranking among the lowest (Garcia Rodriguez 2001). A further randomized controlled trial of ibuprofen, paracetamol or a combination tablet in adults with osteoarthritis showed that paracetamol 3 g/day may cause similar degrees of asymptomatic blood loss (as measured by a drop in haemoglobin), as ibuprofen 1200 mg/day and the combination of the two agents appeared to be additive (Doherty et al. 2011).

The evidence in adults suggests that at over-the-counter (OTC) doses, symptomatic GI side effects with ibuprofen are comparable with placebo, and treatment is well tolerated and largely free of gastric damage (Bjarnason 2013). While there are fewer data regarding GI effects in febrile children, in one of the largest trials of ibuprofen and paracetamol, the risk of GI bleeding was low (7.2 per 100,000), with no statistically significant difference in GI bleeding between the two treatment groups (*p* = 0.31). The four cases of GI bleeding reported in this study occurred in children previously treated with ibuprofen; all were managed conservatively with no endoscopy being required (Lesko and Mitchell 1995a, b). This finding is occasionally cited as a potential cause for concern, despite the lack of significance relative to paracetamol. However, since this early study, other studies have confirmed that (UGIC’s) are rare events in children treated with NSAIDs, with a low absolute risk of about 2.4 UGIC incidents per 10,000 children presenting to emergency departments (Grimaldi-Bensouda et al. 2010; Bianciotto et al. 2013). Of these, minor gastrointestinal side effects are the most commonly reported in clinical trials raising concerns regarding potential adverse gastrointestinal effects with the use of NSAIDs generally.

In addition, in their case–controlled study of children admitted to hospital via emergency departments for acute conditions over an 11-year period, Bianciotto found no significant difference in the risk of UGICs with paracetamol (adjusted OR 2.0, 95% CI 1.5–2.6) compared with ibuprofen (adjusted OR 3.7, 95% CI 2.3–5.9) (Bianciotto et al. 2013), although the adjusted OR for NSAIDs in Grimaldi-Bensouda et al’s study reached 8.2 (95% CI 2.6–26.0), with one-third of cases attributable to exposure to NSAIDs administered at therapeutic doses (although limited dataset and recall bias could not be excluded).

A consequence of the perceived association of NSAIDs and UGIC’s is the common advice to take ibuprofen with food (or fluids such as milk), the rationale being that such co-administration exerts a ‘protective’ effect in the GI tract. This has a particular impact for OTC use in childhood fever, where children may feel too unwell to eat or drink. However, the need for NSAIDs to be taken with food has never been properly studied in humans. It is possible that certain foods may have negative as well as positive effects. Food delays the achievement of peak levels of NSAIDs and so impacts on efficacy, leading to the suggestion that it may be more appropriate to advocate that OTC ibuprofen be taken on a fasting stomach to achieve a rapid onset of action and to avoid the use of an ‘extra’ dose because the speed of action did not meet expectations (Rainsford and Bjarnason 2012). Given that OTC antipyretics are given for a short time period to manage childhood fever, and that rapid onset of action and symptom relief are important aspects, the advice to take ibuprofen with food may not be appropriate for this setting.

### Asthma

Both ibuprofen and paracetamol are commonly used in the management of acute feverish childhood illnesses. The incidence of NSAID- or paracetamol-triggered asthma in children is thought to be less frequent than in adults. In a randomized controlled trial in febrile asthmatic children, those who received ibuprofen were less likely to be hospitalized and significantly less likely to require outpatient visits for asthma compared with children who received paracetamol (Lesko et al. 2002). Debley et al. (2005) investigated the prevalence of ibuprofen-sensitive asthma in 127 children aged 6–18 years with mild-to-moderate asthma. Out of 100 children who completed a bronchoprovocation challenge study, 2 children (prevalence 2%) met the criteria for ibuprofen-sensitive asthma [95% confidence interval (CI) 0.2, 7.0].

Aspirin-induced asthma (AIA) is a well-recognized and distinct clinical syndrome of asthma and rhinitis affecting up to 5% of asthmatic children aged 10 years and older, peaking around the third decade of life. Symptoms of asthma and rhinitis occur about 30 min to 3 h after ingestion of aspirin or other NSAIDs. Episodes of asthma are severe and can even be life threatening; however, many patients are often unaware of their sensitivity either because they have never taken aspirin or have developed AIA in adulthood after years of apparent tolerance (Babu and Salvi 2004). Therefore, the use of aspirin in both the paediatric and adult asthmatic populations should largely be discouraged.

Although the name would suggest a direct link solely to aspirin exposure, there is cross sensitivity to all classes of NSAIDs, which may be of concern to OTC users who suffer from asthma and those who prescribe it to them. The mechanism of sensitivity is thought to be related to COX-1 inhibition favouring lipo-oxygenase activity, which in turn indirectly causes an increase in the production of leukotrienes and subsequent bronchoconstriction. However, the prevalence and cross reactivity to other analgesics have been difficult to assess due to differences in the trial methods. There is emerging evidence that suggests paracetamol may also contribute to the exacerbation of asthma or asthma-related adverse events, and patients who have AIA can also to be sensitive to paracetamol, albeit less severely (Jenkins et al. 2004).

Despite a theoretical potential association between ibuprofen and asthma, evidence suggests a low risk for asthma-related morbidity associated with ibuprofen use in children (Kanabar et al. 2007). Ibuprofen does not appear to exacerbate asthma in children without a history of aspirin sensitivity and may in fact be associated with a lower risk of exacerbation than paracetamol (Kanabar et al. 2007). A cross-sectional analysis using the Third National Health and Nutrition Examination Survey has shown that paracetamol use in adults was associated with an increased risk of asthma and chronic obstructive pulmonary disease and decreased lung function (McKeever et al. 2005). Increasing frequency of paracetamol use (≥14 days per month) was positively associated with an increased risk of newly diagnosed adult-onset asthma (*p* = 0.006). A further study has shown that the incidence of asthma in infrequent users (<monthly) and monthly, weekly and daily users was 1.06, 1.22, 1.79 and 2.38, respectively (*p* = 0.0002) (Shaheen et al. 2000).

The underlying mechanisms for the risk of asthma and impaired lung function have been studied by Eneli and colleagues who have shown that paracetamol decreases the concentration of glutathione, which results in the vulnerability of the asthmatic patient to oxidative stress (Eneli et al. 2005). Low levels of glutathione lead to the inability to counteract oxidative stress and a defective processing of disulphide bonds that are key in antigen presentation. Furthermore, lack of inhibition of COX-2 by paracetamol and the subsequent synthesis of PGE2 promote immunological activity with a T-helper cell type 2 response that establishes an allergic tendency in the immune response to various stimuli.

There is growing concern regarding a potential link between paracetamol and lung injury, bronchoconstriction and asthma development, particularly due to maternal use of this analgesic during pregnancy. Frequent paracetamol use (most days or daily) during late pregnancy (20–32 weeks) was associated with an increased risk of wheezing in the offspring at 30–42 months compared with paracetamol non-users (Shaheen et al. 2002).

Paracetamol use has also been implicated in asthma development and the increasing incidence of asthma in adults and children in epidemiological, observational and pathophysiological studies (reviewed in McBride 2011; Farquhar et al. 2010; Eneli et al. 2005; Beasley et al. 2008, 2011). and more recently in a prospective birth cohort study (Kreiner-Moller et al. 2012).

Although it is difficult to prove a causal pathway between paracetamol use and subsequent long-term conditions, some experts now state that there is ‘overwhelming evidence’ of a link between paracetamol and asthma (Holgate 2011) and have warned about the potential for paracetamol intolerance in some asthmatic patients (Eneli et al. 2005; McBride 2011). Given the widespread use of paracetamol in children, there has been a call for causation to be proved or disproved in adequately powered placebo-controlled trials (Holgate 2011), and clearly more research in this field is required.

In conclusion, evidence suggests that the risk of asthma-related morbidity with ibuprofen use is low in febrile children. The therapeutic benefit of ibuprofen as an antipyretic and analgesic outweighs any putative risk of acute bronchospasm in children with asthma (Kauffman and Lieh-Lai 2004). Furthermore, regular use of ibuprofen has been associated with better lung function (McKeever et al. 2005).

### Cardiovascular effects

The use of certain COX-2 inhibitors was associated with an increased risk of adverse cardiovascular events in adults, leading to a withdrawal from the market (Conaghan 2012). However, cardiovascular events associated with COX-2 inhibitors have not been reported in children (Foeldvari et al. 2009).

### Unintentional ingestion of ibuprofen

Between 2001 and 2008, more than 22,000 children aged 5 years or under visited US emergency departments for ingestion of over-the-counter analgesics other than paracetamol (Bond 2012).

Between 2010 and 2013, ibuprofen accounted for 16% of US emergency department visits for unsupervised over-the-counter liquid medication exposures in young children. (Lovegrove et al. 2015).

In Spain, 38 out of 2157 cases of childhood poisoning were from ibuprofen (Mentegi et al. 2006).

Reports of complications following ibuprofen overdose, particularly in children, are rare. The vast majority of individuals who overdose on ibuprofen alone have no, or only mild, symptoms (Volans et al. 2003). Fatal overdose in adults is extremely rare and generally related to complicating factors such as the presence of other drugs. Cases of symptomatic overdose in children have been reported following ingestion of over 440 mg/kg (Hall et al. 1986), but in general the risk of serious complications following ibuprofen overdose is low (Argentieri et al. 2012).

### Hepatotoxicity and risk of overdose with paracetamol

One of the main concerns with regard to paracetamol use is hepatotoxicity due to overdose. With therapeutic dosing, paracetamol is predominantly metabolized by conjugation with sulphate and glucuronide. Approximately, 5–10% of the drug is oxidized by CYP450-dependent pathways (mostly CYP2E1 and CYP3A4) to a toxic, electrophilic metabolite, *N*-acetyl-*p*-benzoquinone imine (NAPQI) (Corcoran et al. 1980). NAPQI is detoxified by glutathione and eliminated in the urine or bile. The NAPQI that is not detoxified may bind to hepatocytes and produce cellular necrosis. Usually, because of the relatively small amount of NAPQI formed and the adequate supply of glutathione, paracetamol has an excellent safety profile.

A threshold paracetamol dose associated with hepatic toxicity in children has been difficult to establish because of inaccurate recollection of the ingested dose, doses administered during several days and prolonged release products. Rumack and Matthew in their landmark 1975 study did not indicate a minimum dose for toxicity, but emphasized prolongation of the half-life of paracetamol from liver toxicity (Rumack and Matthew 1975). The Rumack and Matthew nomogram of paracetamol levels and time after dose was developed for prediction of risk in acute intoxications, so that a low level does not eliminate the possibility of toxicity caused by chronic ingestion of paracetamol.

Reports of liver toxicity in paediatric patients have suggested that a minimal, single paracetamol dose of 120–150 mg/kg of body weight may be associated with hepatoxicity (Henretig et al. 1989; Alander et al. 2000).

Hepatotoxicity with paracetamol at recommended doses (Iorio et al. 2013; Savino et al. 2011; Ferrajolo et al. 2010) and in the setting of an acute overdose (Heubi et al. 1998; Hameleers-Snijders et al. 2007; Mahadevan et al. 2006) has also been reported in children, and there is significant concern over the possibility of paracetamol-related hepatitis due to chronic overdose following either the administration of supratherapeutic doses or too frequent administration of appropriate single doses (Kubic et al. 2009; Sullivan and Farrar 2011; Rivera-Penera et al. 1997).

Heubi et al. reported a mortality rate of 55% in 47 cases, with half the deaths in children aged less than 2 years. It was determined that the dosage administration ranged from 60 to 240 mg/kg/day administered for between 1 and 42 days (Heubi et al. 1998). The conclusion based on this and other investigations is that paracetamol can cause serious hepatotoxicity in children administered dosages as low as 125–150 mg/kg/day when taken for 2–4 days (Rivera-Penera et al. 1997) with accumulation of the drug being a possible cause (Nahata et al. 1984).

Kearns et al. have suggested that a child susceptible to toxicity is likely to be less than 2 years of age, has been taking 90 mg/kg/day or more of paracetamol for more than 1 day and who is acutely malnourished and dehydrated as a consequence of vomiting, diarrhoea or decreased fluid and nutrient intake (Kearns et al. 1998). It is suggested that in this combination of factors, the mechanisms for detoxification of the hepatotoxic metabolite of paracetamol are more likely to be deficient.

In addition, a recent UK study found that, even when administered under current instructions, underweight children are at risk of receiving approximately twice, and average weight children up to 133% of, the recommended single and cumulative daily dose of paracetamol; this has led to recently proposed changes in dosing recommendations (Eyers et al. 2012a, b). Two reports of hepatotoxicity in association with dosages reported to be in the therapeutic range (Heubi et al. 1998; Makin et al. 1995) may represent inaccurate memory of the administered doses or a narrower paracetamol therapeutic window because of associated conditions. Such conditions might include inherited differences in hepatic enzyme activity, malnutrition, ethanol ingestion, drug interactions or concomitant medical disorders.

It is important to remember that children with a family history of hepatic toxicity to paracetamol have an increased risk of developing a toxic reaction. The health-care provider should consider paracetamol toxicity in any child who has received paracetamol with signs of acute hepatic dysfunction, even if paracetamol levels are not in the toxic range. If the levels are in the toxic range after long-term treatment with paracetamol, it is an ominous finding associated with a high risk of mortality.

Finally, clinical signs of liver disease, such as fever or abdominal pain, are often treated with paracetamol. Whether hepatic injury from underlying conditions, such as viral infections or metabolic diseases, is exacerbated by paracetamol remains uncertain. Many reported cases of severe hepatotoxicity in children have been attributed to cumulative toxicity from repeated doses rather than acute intoxication from a single massive overdose.

### Renal effects

NSAIDs have been associated with the development of acute kidney injury, which is thought to be related to COX inhibition leading to changes in haemodynamics and acute interstitial nephritis (AIN). While it is unlikely that prostaglandins have much impact on children with normal circulating volume, in individuals with volume depletion, their role as vasodilators becomes more important to maintain adequate renal perfusion. Blocked prostaglandin synthesis leads to unchecked vasoconstriction of the afferent arteriole, resulting in reduced GFR and eventually renal ischaemia and acute tubular necrosis (Lameire et al. 2005; Taber and Mueller 2006).

There were no incidences of acute renal failure in a large practitioner-based population study which included 55,785 children treated with ibuprofen (Lesko and Mitchell 1995a, b) or in the Boston Collaborative Fever study which included 27,065 febrile children randomized to ibuprofen (Lesko and Mitchell 1999). A further study by the same author found that, with short-term use of ibuprofen, the risk of less severe renal impairment is small and not significantly greater than with paracetamol (Lesko and Mitchell 1997).

Similarly, a large-scale paediatric study by Ashraf and colleagues found no incidences of renal conditions in over 31,000 children treated with either ibuprofen or paracetamol (Ashraf et al. 1999). There have, however, been rare case reports of reversible renal insufficiency in children with febrile illnesses treated with ibuprofen or other NSAIDs, largely associated with volume depletion (Krause et al. 2005; Moghal et al. 2004; Ulinski et al. 2004). Dehydration is a known risk factor for NSAID-induced acute renal failure, and this has led some experts to recommend caution in ibuprofen use in children with dehydration or pre-existing renal disease (Sullivan and Farrar 2011; Chiappini et al. 2012). Recently, a retrospective chart review of 1015 children with acute kidney injury (AKI) managed over an 11.5-year period concluded that 27 cases (2.7%) were associated with NSAID use (predominantly ibuprofen), and that younger children (<5 years of age) were more likely to require dialysis or admission into ICU (Misurac et al. 2013). This retrospective study raises obvious concerns; however, it has a number of important limitations which make these conclusions questionable. Most importantly, patients with a history of volume depletion, an independent risk factor for AKI, were not excluded from the analysis. The most common presenting symptoms given for children in this study were vomiting and decreased urine output, and the majority of patients defined as having NSAID-associated AKI had a history of volume depletion. It is unclear whether these dehydrated patients may have developed AKI irrespectively of NSAID use.

In clinical practice, the author’s experience is that renal problems arising out of short-term usage (i.e. less than 7 days) of ibuprofen in feverish children are an unlikely occurrence; nevertheless, caution (and common sense) should be applied when administering any agent that may interfere with renal function in a volume-depleted or multi-organ failure child.

Rarely, patients may also present with manifestations of systemic hypersensitivity reactions. Children receiving NSAIDs may also present with nephrotic syndrome (Perazella and Markowitz 2010; Alper et al. 2002). Preventative strategies should include avoiding NSAIDs and/or monitoring those at high risk, including children with volume depletion, pre-existing renal disease or concomitant use of other nephrotoxic drugs.

### Soft tissue infections

Several reports have suggested an association between severe soft tissue superinfection and the use of NSAIDs. In particular, ibuprofen was implicated when its use in children with varicella was linked with the subsequent development of invasive Group A streptococcal infections (Wattad et al. 1994; Petersen et al. 1996).

This concern prompted a retrospective cohort study in which data from over 7000 children with varicella were examined (Choo et al. 1997). This study showed that 89 superinfections developed among 7013 cases of varicella. Out of 169 children receiving ibuprofen within 180 days of varicella diagnosis, only 4 developed a superinfection (OR 1.5 (95% CI 0.3–4.9)). Compared with children who were naive to ibuprofen, those who were dispensed ibuprofen in the month prior to varicella were 3.1 times more likely to be diagnosed with a superinfection (95% CI = 0.1–19.7; *p*= 0.31). This study concluded that the infrequent dispensing and skin superinfection significantly limited the power of the study and that over 54,000 children with varicella would have to be studied to detect a relative risk of 2 with *α* = 0.05 and 80% power.

## Conclusions

Despite the widespread use of ibuprofen and paracetamol, thankfully the rate of severe toxicity in children remains rare. Meta-analyses confirm that the safety and tolerability profiles of paracetamol and ibuprofen in managing children’s pain and fever are similar, and that both drugs are associated with specific rare adverse events, which are difficult to detect and quantify in all but the largest clinical trials.

However, despite published evidence to the contrary, globally paracetamol is perceived as having better GI, renal and respiratory safety and overall better tolerability than ibuprofen. In turn, ibuprofen is perceived as having better GI safety than aspirin and its safety profile, particularly for short-term use is poorly understood among health-care practitioners and the public. This is likely due to the following factors:

1. A lack of distinction from other NSAID’s, resulting in a “class effect” bias.
2. Ingrained negative perceptions from the time of undergraduate or postgraduate training.
3. Lack of awareness of the impact of the dose (Rx versus OTC).
4. A misconception that ibuprofen needs to be taken with food and a perceived gastro protective effect of food—whereas the opposite is true, in that administration with food slows the speed of absorption and may interfere with its efficacy.
5. It is also possible that newly qualified HCPs may be aware of the current state of knowledge with regard to ibuprofen safety and tolerability, but may lack the confidence to put this knowledge into practice—and this needs further exploration.
6. Finally, patients (and parents of young children) tend to display medication-sparing behaviour and have little understanding of the impact of delayed treatment with suboptimal doses on analgesic efficacy.

The aim of this review has been to assess the evidence with respect to clinical and safety factors which would aid HCPs in recommending one agent over another when advising parents of children with pain and/or fever, and the author’s conclusion is that for short-term usage (i.e. for less than 7 days), both paracetamol and ibuprofen have equally good safety and tolerability profiles, and when efficacy data is considered alongside safety, ibuprofen may be more preferable in providing relief from discomfort, fever and pain.
